# The relationship between lymphocyte subsets, nutritional status and tuberculin reactivity in continuous ambulatory peritoneal dialysis and hemodialysis patients

**DOI:** 10.1007/s11255-020-02467-1

**Published:** 2020-04-22

**Authors:** Mehmet Usta, Alpaslan Ersoy, Yavuz Ayar, Ferrah Budak

**Affiliations:** 1 Department of Nephrology, Bursa City Hospital, Nilüfer, 16010 Bursa, Turkey; 2 Department of Nephrology, Faculty of Medicine, Uludag University, Bursa, Turkey; 3 Department of Microbiology and Immunology, Faculty of Medicine, Uludag University, Bursa, Turkey

**Keywords:** Hemodialysis, Continuous ambulatory peritoneal dialysis, Tuberculin testing, Human erythropoietin, Nutritional status

## Abstract

**Aim:**

Skin test anergy is common in patients with uremia and during maintenance hemodialysis treatment. However, up to date only one study concerning skin test in peritoneal dialysis patients has focused on the issue. Our cross-sectional controlled study was conducted to analyze the correlation of purified protein derivative (PPD) test response with demographical features, nutritional parameters and the distribution of peripheral blood lymphocyte subsets in peritoneal dialysis and hemodialysis patients

**Patients and methods:**

Stable 30 hemodialysis (HD) patients (16 men, 14 women) and 30 continuous ambulatory peritoneal dialysis (PD) patients (17 men, 13 women) were included. Thirty healthy cases (15 men, 15 women) with a mean age of 32.4 ± 9.4 constituted the control group.

**Results:**

In the HD group, 14 patients (46.6%) were PPD positive, and ın the PD group 16 patients (53.3%) were PPD positive. In the PPD-positive HD patients 64.2% (9/14), and in the PPD-positive PD patients 62.4% (10/16) had an induration of 10 mm or greater. In the control group, 21 of 30 patients (70%) were PPD positive. Comparison of both HD and PD groups with the control group showed significant differences in PPD reactivity (*p* < 0.01). Albumin levels were significantly high in the control groups (*p* < 0.01), and cholesterol levels were significantly high in the PD and the control groups (*p* < 0.05). Transferrin levels were significantly high in the PD (*p* < 0.01). The lymphocyte counts were significantly high in the control group compared to the HD patients (*p* < 0.05). The lymphocyte subset percentages CD19 were high in the control groups (*p* < 0.05), and CD16/56 was significantly high in the PD groups (*p* < 0.05). All the parameters were also similar between PPD-positive and -negative same groups.

**Conclusion:**

The prevalence of PPD positivity was lower in the PD and HD groups. The PPD test responses were not related to the peripheral lymphocyte counts, subsets and malnutrition parameters.

## Introduction

It is known that an immune deficiency state is observed in chronic hemodialysis (HD) patients due to unknown mechanisms [1]. Both humoral and cellular immune systems have functional impairment in this setting. However, the major defect is generally detected in the cellular immune system in which T-lymphocytes have an important role [2]. Defective cell-mediated immunity, as reflected by anergy to multiple skin test antigens, is well documented in patients with protein-calorie malnutrition [3]. Skin test anergy is also common in patients with uremia and during maintenance hemodialysis treatment [4]. However, the reported prevalence varies widely. The mechanism leading to cutaneous anergy in chronic renal failure remains undefined. Various T cell abnormalities have been demonstrated, but the data are conflicting and there are almost equal numbers of reports of normal and depressed T cell function. The whole subset is beyond the scope of this communication [5–7]. In HD patients, decreased response to tuberculin, which is an anergic skin test, has been described. Simirnoff et al. reported an increased rate of anergy and tuberculin nonreactivity in this group of patients [8]. Recently, Yıldız et al. reported the relationship between tuberculin response and patient demographic features, nutritional parameters, and the distribution of peripheral blood lymphocyte subgroups in HD patients [9]. In this report, they believed that the tuberculin response in hemodialysis patients could not be predicted by quantitative analysis of peripheral blood lymphocyte subtypes. The differences in tuberculin reactivity might originate from qualitative differences in lymphocyte subsets or differences in the dermal in situ immune response.

In the study, we aimed to investigate the association between tuberculin response and patient demographic features, nutritional parameters, and the distribution of peripheral blood lymphocyte subgroups in HD and PD.

## Patients and methods

Stable 30 HD patients (16 men, 14 women) and 30 PD patients (17 men, 13 women) were included in our study. The mean ages were 34.0 ± 12.6 and 34.4 ± 12.6 in the HD and PD patients, respectively. A control group of 30 healthy cases (15 men, 15 women) with a mean age of 32.4 ± 9.4 years were also included. The demographic characteristics, state of erythropoietin therapy and total duration of dialysis are shown in Table 1. In chronic dialysis patients, no additional factors could affect the PPD results, except dialysis when present.

**Table 1:** Demographic characteristics of the patients

	Hemodialysis		Peritoneal dialysis		Control	
	PPD ( +) *n*: 14	PPD (−) *n*: 16	PPD ( +) *n*: 15	PPD (−) *n*: 15	PPD ( +) *n*: 21	PPD (−) *n*: 9
Age (years)	33.9 ± 12.0	34.1 ± 11.8	35.4 ± 13.9	33.5 ± 13.4	31.2 ± 12.6	33.6 ± 10.9
Sex (male/female)	9/8	7/6	10/7	7/6	8/7	7/8
BMI (kg/m^2^)	22.1 ± 3.2	21.2 ± 2.4	23.0 ± 3.0	23.2 ± 3.4	24.0 ± 2.3	23.1 ± 2.9
TSD (months)	30.8 ± 13.8	28.6 ± 14.2	27.0 ± 12.7	28.1 ± 13.7	–	–
Epo (UI/W)	4386 ± 3272	4272 ± 3849	3872 ± 3130	3780 ± 2964	–	–
Primary etiology of renal failure						
Idiopathic	10			9		
Hypertension	5			6		
GN	6			4		
Amyloidosis	3			4		
PKD	2			3		
ATN	1			2		
Pyelonephritis	3			2		

*ATN* acute tubular necrosis, *PKD* polycystic kidney disease, *Epo* erithropoietin, *W* week, *TSD* time spent on dialysis, *GN* glomerulonephritis

### Exclusion criteria

- Presence of diabetes mellitus.
- History of tuberculosis infection.
- HIV positivity.
- Recent viral or bacterial infection.
- Having immunosuppressive therapy.
- A total dialysis duration of less than 6 months.
- Presence of malignancy.
- Age of less than 16 and older than 60 years.

In HD patients, during hemodialysis polysulfone membranes and bicarbonate dialysates were used. HD patients had two or three dialysis sessions per week. PD patients were dialyzed with standard peritoneal fluids containing 35 mmol/L lactate. Serum samples were taken just before the dialysis session and in the biochemistry and immunology laboratory of our center. The serum albumin, cholesterol and creatinine were determined by a standard autoanalyzer method. Serum transferrin was estimated by radial immunodiffusion (Nephelometers). Body mass indexes of all patients were determined. White blood cell count was determined by using Coulter counter and the lymphocyte count was derived from the differential count. PPD solutions were injected to both the patient and the control groups. Kt/*V* was taken as the index of dialysis adequacy, and ın the PD groups weekly Kt/*V* urea was calculated.

### Tuberculin testing

Tuberculin reactivity in HD and PD patients was assessed by response to intradermal 5 IU PPD (Inter Vax Biologicals, Limited, Canada) injected using the Mantoux technique into the volar surface of the forearm and forearm without the arteriovenous fistula in the hemodialysis patients. Induration was measured at 72 h. Indurations of less than 5 mm were defined as PPD negative in the dialysis patients. An experienced medical staff performed the tuberculin testing.

### Peripheral blood lymphocyte subtyping

In the immunology laboratory, immunophenotypic analysis of the cells was performed using an EPICS XL-MCI flow cytometers (Coulter) equipped with a 15 mW air-cooled argon-ion laser. The subgroup of CD3, CD4, CD8, HLA DR^+^ CD3^+^, CD16/56^+^ and CD19 lymphocytes were measured by using monoclonal antibodies in peripheral blood samples. A minimum of 1000 events was counted on each sample. Data analysis was performed using EPICS XL-MCI software (Coulter). Gating was performed using 90^0^ right angle scatter. The fluorescence signals were amplified on a logarithmic scale.

### Statistical analysis

We reported all numerical values as mean ± standard deviation (SD). Statistical computations were done using SPSS for Windows V. 20.0 (SPSS Inc. Illinois, USA). Using the nonparametric Kruskal–Wallis ANOVA test, we did comparisons between groups. Statistical significance was assumed for *p* values less than 0.05.

## Results

The comparison of both dialysis groups revealed no significant differences in mean ages, sex, body mass indexes, and total duration of dialysis (*p* > 0.05). In addition, comparison of dialysis patients with the control group revealed no significant differences in characteristics of patients (*p* > 0.05). Distributions of dialysis patients according to primary etiologies were similar (Table 1).

The mean durations of HD in PPD-positive and -negative patients were 30.7 ± 13.8 and 28.6 ± 14.2 months, respectively (*p* > 0.05). In the PD group, the mean duration of dialysis was 27.0 ± 12.7 and 28.1 ± 13.7, in the PPD-positive and -negative patients, respectively (*p* > 0.05). In all dialysis groups, erythropoietin dosages did not differ significantly (*p* > 0.05).

In the HD group, 14 patients (46.6%) were PPD positive and 16 (53.3%) were PPD negative. In the PD group, 16 patients (53.3%) were PPD positive and 14 (46.6%) were negative. In the PPD-positive HD patients 64.2% (9/14) and in the PPD-positive PD patients 62.4% (10/16) had an induration of 10 mm or greater. In the control group, 21 of 30 patients (70%) were PPD positive. The mean PPD values are demonstrated in Table 2. The PPD response of the two patient groups were compared and no statistical significance was found (*p* > 0.05). However, comparison of both HD and PD groups with the control group showed significant differences in PPD reactivity (*p* < 0.01) (Table 2).

**Table 2:** PPD values and response rates of the groups

	HD	PD	Control
PPD (+) (%)	46.6	53.3	70
PPD (mm)	7.6 ± 5.7	8.2 ± 6.0	16.7 ± 4.8^a^

^a^*p* < 0.01, both dialysis groups were compared to the control group

Serum creatinine levels were determined in all dialysis patients with a Kt/*V* of greater than 1.7 and in the PPD-positive and -negative dialysis patients no significant intra- and intergroup differences were demonstrated (*p* > 0.05). Albumin levels did not significantly differ in the dialysis groups and intergroup comparisons revealed no significant differences (*p* > 0.05). In the control group, albumin levels were significantly high compared to the dialysis groups (*p* < 0.05). Serum cholesterol levels were significantly high in the PPD-positive and -negative control groups and the PPD-positive and -negative PD groups compared to the HD groups (*p* < 0.05). The serum cholesterol levels were uniformly distributed in all groups (*p* > 0.05, Table 3). Transferrin level was higher in PD than HD (*p* < 0.01); however, intergroup comparisons revealed no significant differences (*p* > 0.05).

**Table 3:** Nutritional parameters of the patients

	PPD ( +)HDp	PPD (−) HDp	PPD ( +)PDp	PPD (−)PDp	PPD ( +)C	PPD (−)C
Creatinine (mg/dL)	10.7 ± 2.8	12.2 ± 2.0	11.0 ± 2.7	11.4 ± 3.2	–	–
Albumin (g/dL)	3.6 ± 0.4	3.7 ± 0.2	3.5 ± 0.3	3.6 ± 0.3	4.0 ± 0.3^a^	4.1 ± 0.4^a^
Cholesterol (mg/dL)	151 ± 36	158 ± 47	208 ± 37^b^	183 ± 30^b^	188 ± 49^b^	203 ± 32^b^
Transferrin (mg/dL)	1.61 ± 0.3	1.58 ± 0.3	1.92 ± 0.4^c^	1.82 ± 0.4^c^	–	–

*HDp* hemodialysis patients, *PDp* peritoneal dialysis patients, *C* control

^a^*p* < 0.01, compared to the PPD-positive and -negative dialysis groups

^b^*p* < 0.01, compared to the PPD-positive and -negative HD groups

^c^*p* < 0.01, compared to the PPD-positive and -negative HD groups

In all groups, peripheral lymphocyte counts and subgroups were evaluated. There was no a significant difference between the lymphocyte counts of the HD and PD groups (*p* > 0.05). The lymphocyte counts were significantly higher in the control groups compared to the HD patients (*p* < 0.05). There was no significant difference between those of control and PD groups (*p* > 0.05). The lymphocyte subsets CD3, CD4, CD8, and CD4/CD8 did not change significantly among all groups (*p* > 0.05). However, the lymphocyte subset CD19 was significantly higher in the PPD-positive and -negative control groups compared to both dialysis groups (c:*p* < 0.05, d:*p* > 0.01, Table 4). The subset CD16/56 was significantly higher in PPD-positive and -negative PD groups compared to the control groups and HD groups (*p* < 0.05).

**Table 4:** Peripheral lymphocyte counts and subgroups

	HDp, PPD (+)	HDp, PPD (−)	PDp, PPD (+)	PDp, PPD (−)	C, PPD (+)	C, PPD (−)
PBL, /mm^3^	1518 ± 718	1535 ± 634	1787 ± 653	1826 ± 679	2105 ± 458^a^	2090 ± 409^a^
CD3, %	73.2 ± 8.9	73.3 ± 8.2	68.8 ± 8.7	72.3 ± 6.3	72.9 ± 5.9	70.6 ± 4.3
CD4, %	44.1 ± 8.3	42.8 ± 6.1	45.2 ± 4.9	48.0 ± 5.2	46.4 ± 7.3	42.0 ± 5.4
CD8, %	30.3 ± 7.8	30.2 ± 5.8	25.5 ± 7.4	26.1 ± 5.9	25.5 ± 4.6	25.8 ± 6.0
CD4/CD8	1.6 ± 0.6	1.4 ± 0.3	1.9 ± 0.5	1.9 ± 0.4	1.8 ± 0.5	1.7 ± 0.5
CD19, %	8.4 ± 3.1	8.2 ± 1.9	8.9 ± 5.0	8.5 ± 4.1	11.5 ± 3.9^b^	13.4 ± 5.6^c^
CD16^+^/56^+^,%	7.6 ± 4.2	8.0 ± 5.7	13.5 ± 7.8^d^	13.9 ± 6.6^d^	7.2 ± 4.0	6.3 ± 3.0
HLA DR^+^CD3^+^,%	25.2 ± 12.8	21.5 ± 9.1	26.3 ± 9.9	19.9 ± 8.1	19.9 ± 5.8	22.6 ± 9.9

*PBL* periheral blood lymphocyte, *HDp* hemodialysis patients, *PDp* peritoneal dialysis patients, *C* control

^a^*p* < 0.05, compared to the PPD-positive and -negative HDp groups

^b^*p* < 0.05, PPD( +) C group was compared to the PPD-positive and -negative HDp and PDp groups

^c^*p* < 0.01, PPD(−) C group was compared to the PPD-positive and -negative HDp and PDp groups

^d^*p* < 0.05, compared to the PPD-positive and -negative C groups and HDp groups

## Discussion

Delayed skin hypersensitivity was clearly depressed, out of proportion to the number of patients manifesting protein-calorie malnutrition. Protein-calorie malnutrition had been described in the chronic hemodialysis population. Positive skin tests were seen in 36–50% of the uremic patients. They concluded that the incidence of anergy increased with the duration of dialysis. Delayed cutaneous hypersensitivity to microbial or protein antigens is commonly depressed in uremic patients (50–60%) compared to healthy controls [1, 3–5, 10]. In our study, we found that the prevalence of PPD positivity in chronic HD and PD patients was %46.6 and %53.3, respectively. In PPD-positive HD patients 64.2% (9/14), and in PPD-positive PD patients % 62.4 (10/16) had an induration of 10 mm or more. The PPD positivity rates of these two groups were similar as well as the total dialysis duration. However, both the HD and PD groups had significantly lower PPD positivity rates compared to the control groups. Studies concerning immune system status, nutrition and dermal anergy tests are frequently held in hemodialysis groups, and not much data are available for PD patients. Taskapan et al. evaluated 30 PD patients and found that 20% had indurations of 5 mm or more in dermal PPD anergy test [7]. In our study, serum albumin and creatinine levels, which are important parameters for malnutrition, were similar in PPD-positive and -negative HD and PD groups. Serum cholesterol levels were significantly high in both the PPD-positive and -negative PD groups. The high levels in the PD group may be due to high glucose content in the dialysate solutions and better nutritional status in peritoneal dialysis patients in contrast to other studies. The prevalence of malnutrition in PD patients is greater than that in HD patients. A recent international multicenter study found a prevalence of moderate to severe malnutrition in 40% of these patients [11, 12]. In our study, PPD positivity, albumin and creatinine levels and BMI were similar in the PPD-positive and -negative HD and PD groups. In the control group in PPD-positive and -negative patients, serum albumin levels were significantly high. Albumin is a positive marker for malnutrition, but we considered the high levels of serum albumin to be independent in the PPD-positive control group. Additionally, transferrin that is a nutritional parameter was significantly higher in the PD group. There was no difference in both groups in terms of total lymphocyte count, and PPD positivity and total lymphocyte count were significantly higher in the control group compared to PD and HD patients. Iron overload, due to changes in its levels in proteinuric patients and being an acute phase reactant, is not accepted as an optimal nutritional parameter. Rapid turnover of iron may helps short-term evaluation of nutritional status. In the study ion dialysis patients, the measured PPD reactivity was significantly low. The mechanism leading to cutaneous anergy in chronic renal failure remains undefined. Various T cell abnormalities have been demonstrated, but the data are conflicting and there are almost equal numbers of reports of normal and depressed T cell function. Uremic patients are always profoundly lymphopenic [5, 6, 13]. Significantly low absolute counts of the total population of T cells as well as T cell regulatory subsets and B cells have been described [14]. Uekı et al. reported that the numbers of peripheral blood lymphocyte, CD3, CD4, CD8, and CD20 cells were decreased in HD patients compared to those in healthy subjects, while the number of CD3 HLA-DR cell was increased in HD patients compared to that in healthy subjects. In this study, they documented that HLA DR CD3 levels decreased after 1 month of erythropoietin therapy and gradually declined throughout the 6-month study period [15]. In the PD patients, the previously mentioned parameters are not fully evaluated and not much data comparing PPD-positive and -negative HD and PD groups are available. In our study, the total lymphocyte counts were similar in the PPD-positive and -negative HD and PD groups. However, in the PPD-positive and -negative HD patients, the total lymphocyte counts were significantly lower than that in the PPD-positive and -negative control group. The total lymphocyte counts did not differ between the PPD-positive and -negative groups in the dialysis patients. In addition, in the PD group the total lymphocyte counts were a little higher in the PPD-positive patients. In the control group, high blood lymphocyte count may be associated with PPD positivity. Impaired immune response in uremia may be caused by multiple derangements of the immune system. In another investigation evaluating the lymphocyte subsets. Deenitchina et al. reported that CD3 and CD4 percentage rate was significantly high in HD patients than that in the control group [1]. In another study conducted by Yıldız et al., 29 HD patients were studied and the HLA DR CD3 percentage rate was significantly high in the PPD-positive and -negative HD group than that of the healthy controls. CD3, CD4, CD8, CD4/CD8, CD19 and CD16/56 percentage rated were similar [9]. In our study, the CD16/56 lymphocyte subsets were significantly more frequent in the PPD-positive and -negative PD groups than that of the PPD-positive and -negative HD and the control patients. CD19 percentage rate was significantly high in the control group compared to both dialysis groups. The percentages of subgroup of CD3, CD4, CD8 lymphocytes, and the CD4/CD8 ratio were similar in all groups. The results of our study are in concordance with the ones regarding the HD population, but in PD patients the data on these subsets are not much. In a study, a lowering effect of erythropoietin in lymphocyte subsets of HLA DR CD3 in HD patients was demonstrated [15]. In our study, we did not have data encouraging us to think erythropoietin causes a decrease in lymphocyte subsets of HLA DR CD3. In our study, the distribution of lymphocyte subsets was similar in dialysis patients receiving the same amount of erythropoietin, except for the high prevalence of CD16/56 ratio in PPD-positive and -negative PD patients. The subsets were also similar in the control group, except for the high prevalence of lymphocyte subgroup CD19. In general studies, lymphocyte subgroups are expected to be lower in dialysis patients compared to healthy individuals [1, 9, 15]. In our study, CD19 levels were higher in the control group.

In conclusion, nutritional status, PPD results, and peripheral lymphocyte counts were similar in PPD-positive and -negative patients in all dialysis groups. Lymphocyte subset CD19 was more prevalent in the control group and CD16/56 ratio was more prevalent in the PD group. The other lymphocyte subset distributions did not differ significantly among all dialysis groups and the controls. In addition, PPD skin anergy test and serum albumin levels were high in the control groups compared to the dialysis groups**.** In this study, though the lymphocyte counts were similar in PPD-positive and -negative PD and the control groups, PPD positivity was significantly high in the control group. This may be due to a lymphocyte function abnormality in the PD patients, as reported in HD patients previously [16, 17]. Another interesting point is the similarity of malnutrition parameters, lymphocyte counts and subsets in all same groups between PPD-positive and -negative patients, suggesting that other possible factors influencing the PPD results may exist. Nutritional status, immunity and body resistance, and personal characteristics of dialysis patients (history of disease, etc.) increase the tendency toward infection and may affect the PPD results [18–20]. Our study supports these data. There is no difference in tuberculin and qualitative tests in terms of PPD positivity and negativity for both dialysis types. We need larger studies. In addition, though dialysis patients have an increased risk of mycobacterium infection, PPD skin test is not frequently reactive and cannot rule out a tuberculosis infection.
